# The forgotten pandemic: how understanding cholera illuminated mechanisms of chloride channels in multiple diseases

**DOI:** 10.1172/JCI184297

**Published:** 2024-08-15

**Authors:** Qais Al-Awqati

**Affiliations:** Departments of Medicine and Physiology & Cellular Biophysics, Vagelos College of Physicians & Surgeons, Columbia University, New York, New York, USA.

## The microbial unification of the world

Unbeknownst to many, we are living during the seventh pandemic of cholera in modern times. Descriptions of a cholera-like illness in India go back millennia, but pandemics are counted from occurrences from 1817 to the present. The current cholera pandemic began in 1961 on the island of Sulawesi (Indonesia) and subsequently spread globally. Pandemics are promoted by ease of travel, with the rapid transfer of individuals from one region to another, leading to what the great French historian Emmanuel Le Roy Ladurie famously called “the microbial unification of the world” (1). *Vibrio cholerae* became a link between East and West as well as rich and poor. Since the beginning of this pandemic, modern research has identified new physiological processes that cause the disease and led to the development of a rational treatment. Oral rehydration therapy (ORT) was a direct result of translation of basic research to the treatment of large populations, reducing mortality from 60% or more to effectively zero (2). New oral vaccines were developed that are highly effective. Many of the foundational papers in this effort were published in the *JCI*. Researchers in cholera brought the bench to the bedside, before that term became a cliché. Like many efforts in understanding basic mechanisms that underlie disease, the information gained has not only illuminated the particularities specific to cholera, but identified new physiologies and pathologies of several other important organs and maladies.

## The seventh cholera pandemic

The causative agent of the current pandemic is *Vibrio cholerae* El Tor — a somewhat hardier organism than classical *V*. *cholerae*, but they both require higher salinity to grow, and humans are the only reservoir. Contaminated water used for drinking or washing food is the culprit in its spread, but unlike recent viral pandemics, person-to-person spread is rare, with no transfer of infection to medical personnel who take care of patients. This pattern of transmission is likely due to the sensitivity of the organism to gastric acid. In studies of healthy prisoner volunteers, diarrhea did not develop below an ingested dose of 1 × 10^8^ organisms and cholera-like diarrhea required 1 × 10^11^, yet cholera began to appear at a dose of 1 × 10^5^ when the bacteria were given with NaHCO_3_ (3). (For a review of the vexed and contentious problem of using prisoners in medical research, see ref. 4, where one will find that the cholera study would very likely be approved today).

In the present pandemic, no continent has escaped cholera; it reached the Middle East in 1964, Africa in 1971, and the Americas in 1991. Outbreaks continue to appear to this day (WHO Global Cholera and Acute Watery Diarrhoea Dashboard, https://who-global-cholera-and-awd-dashboard-1-who.hub.arcgis.com/), often in regions of conflict. In 1966, cholera struck Baghdad (Iraq), causing panic in the population (5). The Ministry of Health repurposed a tuberculosis hospital for the treatment of cholera, and the Department of Medicine in the university hospital where I was the senior medical resident was asked to staff the isolation hospital with junior residents, medical students, and two senior physicians. We were lucky that my mentor, Mahmoud Thamer, a Johns Hopkins–trained physician, became the chief of the service, and his leadership made us aware of the recently published literature on the modern treatment of cholera diarrhea with intravenous fluids. We had stellar success and less than 1% of our hundreds of patients died, all three of whom had significant comorbidities (5).

## The pathophysiology of cholera and the discovery of intestinal secretion

Until the 1960s, the dominant idea of how cholera causes diarrhea was based on a 19th century model by Virchow who concluded that the diarrhea resulted from transudation or exudation of fluid from the circulation, based on observing desquamation of the intestinal epithelium in autopsies. Research performed by scientists in the United States and Southeast and South Asia provided the first inkling of the pathophysiology; electrolyte composition of the stool output was measured and found to be protein free as well as iso-osmotic with plasma, with higher K^+^ and HCO_3_^–^ concentrations (6). These findings provided the basis for a rational composition of intravenous hydration fluids. Yet even in this landmark *JCI* paper (and despite the difference in composition between stool and plasma), the idea that this fluid was a transudate was considered a possible cause of the diarrhea. But when the clearance of small molecules like mannitol was found to be very low, transudation as a cause was no longer tenable (7).

Another proposed cause of the massive amounts of fluid in the diarrhea was inhibition of intestinal absorption (6). At that time, the modern study of ion transport across epithelia had just begun, pioneered by Hans Ussing who placed frog skin in a chamber that separated the apical from the basolateral media, which allowed for voltage clamping and the study of unidirectional isotope fluxes. With Ussing’s help, a culture filtrate of *V*. *cholerae* was found to contain a heat-labile factor that inhibited the membrane potential of the frog skin, a marker of sodium absorption, strengthening the idea that cholera diarrhea might be due to inhibition of absorption (8). Remarkably, the idea that the intestinal epithelium itself might be the source of the fluid was not yet an appreciated aspect of gastrointestinal physiology. The stomach, pancreas, and biliary tree were known to secrete fluid, but not the intestinal epithelium. By a happy coincidence, William B. Greenough attended a seminar given by Michael Field, a young assistant professor just starting his independent career, who had developed an Ussing-type method to study intestinal transport. Field presented data to show that cyclic AMP induced chloride secretion across the ileum in the absence of electrochemical gradients (9). Following the seminar, it was obvious to both that cholera toxin might be doing similar things and what was needed was a willing person to do the studies. I was then a resident at Baltimore City Hospital and my mentor, the chief of medicine Julius Krevans, encouraged me to work with Greenough. I was the lucky recipient of not only mentorship, but also lifelong friendship of Greenough and Field. After a period of training in Mike Field’s lab in Boston where I was inducted into the ins and outs of ion transport across epithelia, I set up the equipment at Johns Hopkins in Greenough’s lab. Studying isotope fluxes of Na^+^ and Cl^–^ across the short-circuited rabbit (and later human) ileum, we discovered that cholera exotoxin induced chloride secretion, and that this process was the same as that induced by cyclic AMP. Furthermore, we found that Na^+^-coupled glucose transport was preserved in cholera toxin–treated epithelia (10). There was also a “residual ion flux” later identified to be HCO_3_^–^ secretion. Field (and two other groups) discovered shortly thereafter that cholera toxin stimulated adenyl cyclase, increasing cyclic AMP levels in the intestine (11–13). Furthermore, cholera toxin was found to be an ADP-ribosyl transferase that covalently modified the α subunit of G proteins, which was the direct effect that caused the toxin to stimulate adenyl cyclase (14).

## Secretory diarrheas other than cholera

Finding the origin of the diarrhea in cholera not only established intestinal secretion as a physiological phenomenon, but introduced the concept of secretory diarrhea. It was well known that there were many syndromes of explosive diarrhea, often endemic and frequently affecting tourists, that were associated with *E*. *coli*. We tested culture filtrates of enterotoxigenic *E*. *coli* and found that it also induced Cl^–^ secretion, very much like the effect of cyclic AMP (15). The enterotoxin was later purified, and Michael Field discovered that its heat-stable moiety caused Cl^–^ secretion through stimulation of guanylate cyclase, increasing cyclic GMP rather than cyclic AMP (16). But other bacteria that cause travelers’ diarrhea such as *Yersinia* and *Salmonella* and a heat-labile *E*. *coli* toxin increase cyclic AMP. Similarly, endocrine tumor syndromes such as vasoactive intestinal peptide–producing tumors and others induce secretion via cyclic AMP or increased intracellular calcium. Drugs that activate cyclic AMP (prostaglandins) or guanylate cyclase are now in clinical use for the treatment of chronic constipation.

## Intravenous and oral rehydration

Intravenous fluid therapy, an emblem of modern medicine, was invented for the treatment of cholera in 1830 (17; see ref. 18 for an extensive review). Appropriate intravenous therapy based on measurement of the composition of the stools lost and its reflection in the acid base and electrolyte disorders was established 130 years later when Phillips and his group presented their results in the *JCI* in 1959 (6). In those days, before disposable intravenous equipment, needles and tubes had to be individually sterilized. The provision of sterile solutions required significant technical resources difficult to produce in bulk under epidemic conditions. It was thus evident to most workers in the field that the ideal therapy would be oral replacement.

In 1962, Robert Crane demonstrated that active transport of glucose required the presence of Na^+^ (19) and suggested that Na^+^ moved with glucose. I had just finished medical school and my physiology mentor excitedly told me about Crane’s paper as an example of a revolutionary concept in epithelial physiology. During the cholera epidemic, we decided to use this information for oral rehydration and produced fluids that contained saline with added glucose. Patients were first stabilized with intravenous hydration and were moved to a separate ward where oral hydration was provided (5). We did not document the composition of the oral fluid or the response of the patients, though anecdotally everyone did well. About the same time, two other groups, more scientifically versed than we were, provided the critical pieces of information where the composition was designed to allow ingestion of large volumes of fluid (20, 21). Ruxin has written a valuable review of the development of oral rehydration therapy (ORT) based on interviews of all participants in this effort (22). Further developments ensued with the use of digestible macromolecules that helped reduce the osmolarity of the ingested fluid and accomplished equivalent levels of rehydration (23). ORT was also useful in other diarrheal diseases, including those produced by toxigenic *E*. *coli*, rotavirus, and other organisms. The establishment of the International Centre for Diarrhoeal Disease Research, Bangladesh (now known as ICDDR,B), led by my mentor, William Greenough, brought sophisticated scientific research to areas of the world lacking in them and helped train generations of local scientists in the use of this treatment (2). ORT has saved millions of lives through many such efforts all over the world.

## Cyclic AMP–induced chloride secretion: a general principle

The discovery of cholera-induced, cyclic AMP–mediated chloride secretion led to a search for a similar process in other epithelia and was next found in the trachea (24). Later, several other epithelia such as the pancreatic duct, seminiferous tubules, corneal, conjunctival, and nasal epithelia were found to secrete anions (often HCO_3_^–^) in response to hormones that induced cyclic AMP generation. Surprisingly, epithelia lining the cysts in polycystic kidney diseases also secrete chloride in response to cyclic AMP produced by vasopressin, a process that had led to a useful treatment by blocking the vasopressin V2 receptors.

The steps by which chloride moves from blood to lumen against electrochemical gradients were not evident initially, but we found that it was tightly linked to the presence of sodium, since removal of Na^+^ completely blocked it (24). Another link to Na^+^ transport was the finding that it was inhibited by the diuretic, ethacrynic acid, whose target was Na^+^ absorption in the loop of Henle (25). But the details of the cellular model had to await the elegant studies of Welsh and Frizzell using electrophysiological and ion-sensitive electrode measurements. They found that Cl^–^ entered the cell from the basolateral side in a Na^+^-coupled manner and exited the apical membrane through a cyclic AMP–activated Cl^–^ channel (26). The molecular basis of these transport proteins was later identified, with the basolateral transporter being the Na^+^-K^+^-2Cl^–^ cotransporter (NKCC, the target of loop diuretics) and the Cl^–^ channel being the cystic fibrosis transmembrane regulator (CFTR).

## Cystic fibrosis: the inverse of cholera

It became rapidly evident that chloride secretion was at the center of the pathophysiology of cystic fibrosis (reviewed in ref. 27). A chloride channel was present in the apical membrane of trachea that was opened by cyclic AMP in normal, but not in cystic fibrosis, epithelia. Once the gene for CFTR was identified, rapid progress in the field showed that the major mutation, ΔF508, caused the channel to be retained in the endoplasmic reticulum, but if it was induced to move to the apical membrane it could function, although at a reduced level. These exciting discoveries have led to the development of several generations of drugs by Paul Negulescu, now in widespread clinical use, which have revolutionized the treatment of cystic fibrosis; some of the drugs open the CFTR, while others act as chaperones, helping it to leave the endoplasmic reticulum and go to the apical membrane (28).

## Coda: pandemics become “endemicized”

The response of the world to a pandemic often begins with panic and irrational behavior. I still remember that in 1966 some European countries refused to take mail from Iraq for fear of transmission of the contagion. But this heightened anxiety soon gets replaced with efforts to deal with the consequences of the infection in a more studied manner. A question remains, however, as to why cholera receded from the public imagination. Is it that it affects faraway people who are poor or are under the stress of wars? The likely reason, I think, is that pandemics become “endemicized.” The microbe that had once unified our world receded into the background and became yet another disease that we must deal with since, despite the presence of effective treatment, infections will continue to occur. We are living now through this phase with COVID-19. Cholera continues to cause up to 4 million cases a year, with fatalities as high as 140,000 despite the effectiveness of ORT. It is very hard to talk about the impact of ORT on world health without descending into hyperbole. However, the provision of enough packets of ORT salts and instructions for its proper dilution, and in the distribution and education of population in remote areas remain challenging. The use of oral rehydration in the home is the obvious and ideal target of therapy, but it is underutilized and underresourced (29). Compounding all of this is the primary problem of provision of clean water. The reason cases continue to occur is a clear indication that the domain of the struggle has shifted from the laboratory and the clinic to that of implementation, including delivery of ORT packets and information to the region. That requires different expertise and different modes of thinking and societal and political commitments beyond medical expertise.

## Version 1 08/15/2024

Electronic publication
